# Novel phase distributions for large electronically beam-scanning reflectarrays

**DOI:** 10.1038/s41598-021-00883-6

**Published:** 2021-11-08

**Authors:** Ahad Sheikholeslami, Zahra Atlasbaf

**Affiliations:** Department of Electrical and Computer Engineering, Tarbiat Modares University, Tehran, Iran

**Keywords:** Electrical and electronic engineering, Computational science

## Abstract

In this paper, the hybrid combination of genetic algorithm and particle swarm optimization (GAPSO) is used to optimize the phase distribution (PD) of beam-scanning reflectarray. The GAPSO takes advantage of both conventional algorithms and it could cover their weaknesses. Two novel PDs are proposed in this paper which constant phase elements (CPEs) and ordinary elements (OEs) are two basic kinds of elements used in them. The phases of CPEs are fixed and it is not changed during beam scanning and only OEs’ phase could be adjusted to scan the main beam. In this work GAPSO and two novel PDs are applied to array factor’s PD of a 30 × 30 reflectarray antenna to displace the main beam electronically in the vertical plane from − 40° to 40°. Also, in these two novel PDs, 28.8% of total elements are selected as CPEs. In the first one with only CPEs, the phase of OEs (71.2% of total elements) could adjust, but in the second novel PD with CPEs and phase symmetry plane 35.5% of the total elements’ phase could be changed to scan the beam. Optimization results show that the novel PD and hybrid algorithm have appropriate performance in the electronically beam scanning of reflectarrays.

## Introduction

Microstrip reflectarrays are a combination of reflectors and phased arrays. Because of their advantages over parabolic reflectors and phased arrays, they have attracted many researchers’ attention in the last decade^1–4^. Microstrip reflectarrays consist of a printed array that acts as a reflector and a space feed pointed at the focal point^5^. The Beam-scanning capability of reflectarrays is the most important feature in wireless systems like satellites and radars^6^. Feed tuning and aperture phase tuning are the two major methods that have been utilized for beam-steering in reflectarrays. To get the beam in the desired direction by using the feed tuning method, the phase center of the feed antenna must be displaced and there is no need to change reflector surface features. On the other hand, in the aperture phase tuning method, the phase of each radiating element must be adjusted to achieve the desired beam direction^7^. To scan the main beam electronically, there are two different ways of adjusting the phase of elements or phase distribution on reflectarray: (1) computing element phase analytically, (2) Optimizing phase distribution on reflectarray^8^.

The iterative projection method for computing the phase distribution on reflectarray has been proposed in Ref.^9^, where a varactor diode has been used to apply phase changes in the elements. While in Ref.^10^, the nematic liquid crystals and analytically phase distribution have been taken into account to steer the beam. Authors in Ref.^11^ took advantage of RF MEMS to apply phase changes in the elements. The beam-scanning performances of 1-bit pin diode loaded elements are discussed in Ref.^12^. A multi-beam pattern is obtained in Ref.^13^, where the PSO algorithm is employed for optimizing the phase distribution. The quad beam was obtained by using the alternating projection method for phase optimization in Ref.^14^. In Ref.^15^, the phase-only optimization technique (POT) is applied to design the reflectarray with the contoured beam. The PSO and MOPSO were also used to optimize the phase distribution for single reflector bifocal reflectarray for beam scanning in Ref.^16^. In Ref.^17^ a beam scanning reflectarray with PIN diode loaded element is presented, the beam scanning is realized by dynamically tuning the phase shifts of all reflection elements independently. Also, a dual-band beam scanning reflectarray with a PIN diode was proposed in Ref.^18^. Furthermore In Ref.^19^ the novel phase synthesis approach for wideband reflectarray is used to optimize the phase distribution of reflectarray. The phase-matching method (PMM) is used to optimize the phase distribution for mechanically beam scanning reflectarray in Ref.^20^. Also, a contoured beam was achieved using the general framework optimization method in Ref.^21^. In Ref.^22^ Generalized Intersection Approach is used to produce dual-band dual-linear polarized contour beam reflectarray. In recent years, with the advent of 5G, the use of beam-scanning reflectarrays in this field also expanded. Authors in Refs.^23–25^ reviewed the problem and future potential of reflectarrays in 5G applications.

In the above-mentioned research works, for all of the electronically beam-scanning reflectarrays, all of the elements’ phases are optimized or changed to syntheses the beam in different directions. Thus, on electronically reconfigurable reflectarrays, each element needs at least one phase-shifter. For large reflectarrays, this number of phase-shifters and biasing devices for active phase shifters will be very expensive and hard to implement.

In this paper, two novel phase distributions have been proposed to overcome the mentioned drawbacks. To achieve beam-scanning capability in these types of phase distributions, there is no need to optimize or change the phase of all elements and it will be enough to optimize and change phase a few of them. These structures consist of two kinds of basic elements: (1) Ordinary Element (OE). (2) Constant-Phase Element (CPE). A CPE is an element with a constant phase for all scanning values of the main beam while OE is an element that its phase adjusts and changes during beam scanning. The hybrid GAPSO algorithm which is a combination of conventional genetic algorithm (GA) and particle swarm optimization (PSO) has been used for adjusting the phase of OEs in this paper.

This paper is organized as follows: The types and procedures of the GAPSO algorithm are described in detail in “GAPSO algorithm procedure” In “Optimizing setup”, the optimizing setup is described. The reflectarray setup is explained in “Reflectarray setup” and the concept of phase distributions for reflectarray is introduced and optimization results are demonstrated and illustrated in “Novel phase distributions and optimization results”. In “Simulation results” simulation setup and its results were described and Finally, some important conclusions are summarized in “Conclusion”.

## GAPSO algorithm procedure

**Figure 1:**
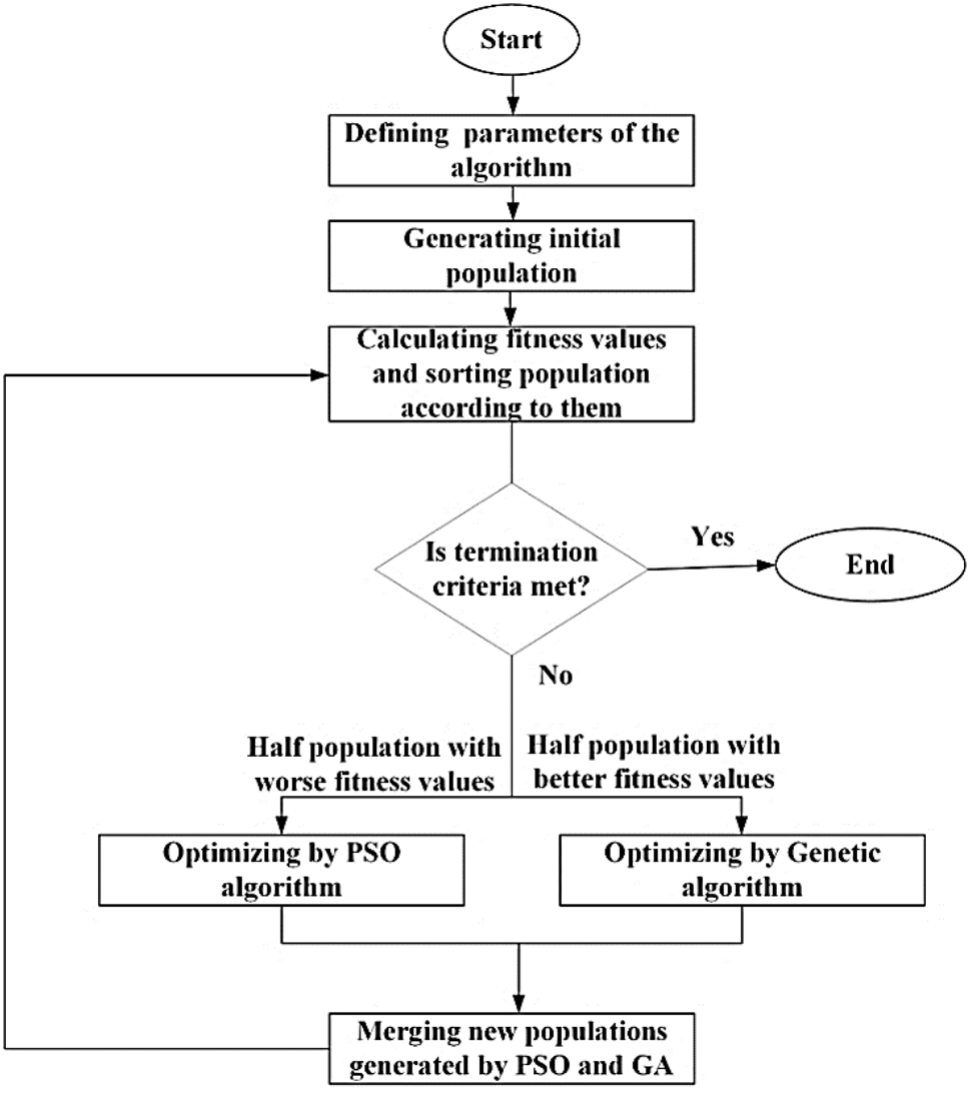
GAPSO algorithm structure.

GAPSO is a combination of GA and PSO algorithms. Various and different combinations of these two optimization methods have been presented in the form of a hybrid GAPSO algorithm so far. In one type of GAPSO algorithms, first, GA is utilized to optimize the problem. Afterward, at the end of the GA process, the obtained results from GA are entered as the initial values to the PSO algorithm and the optimization process is continued by it^26^. Another type of combination method is presented in Ref.^27^. According to this method, the crossover and mutation functions of the GA are used in the PSO algorithm to escape the local optimal points. One of the other GAPSO hybrid algorithms uses GA and PSO in parallel. In this method, half the initial generated population is optimized by GA, and the other half is optimized using the PSO algorithm in parallel^28,29^. In Refs.^26–29^, it is shown that the hybrid GAPSO algorithm is more efficient, reliable, and faster than the conventional GA and PSO algorithms. Hence, in this paper, hybrid GAPSO is applied to optimize the phase distribution on reflectarray for beam-scanning capability. The type of GAPSO that is used in this research work is the parallel GA and PSO. In this method, half of the initial generated population with better fitness values is optimized by GA to avoid local optimal points and to search a wider area. The other half with worse fitness values is optimized by PSO due to the better convergence speed of this algorithm. This type of GAPSO was first used to optimize the phase distribution of the planar beam-scanning reflectarrays to achieve two novel phase distributions for beam-scanning reflectarrays with much lower phase shifter needed for unicells in the reflectarray. The flowchart of a GAPSO algorithm is shown in Fig. 1.

## Optimizing setup

The magnitude of elements in reflectarray antennas is depending on the features of the feed and is usually fixed. So, the only parameter that can be controlled to achieve the beam-scanning performance, is the elements’ reflection phase. As discussed in “Introduction”, optimizing and calculating analytically, are the two approaches for adjusting the phase distribution on reflectarray. The GAPSO explained in “GAPSO algorithm procedure” is used in this paper to optimize the reflectarray array factor phase distribution. Array factor of M*N array is calculated using (1)^30^.

1 $$AF\left(\theta ,\varphi \right)={\sum }_{m=1}^{M}{\sum}_{n=1}^{N}{I}_{m,n}{e}^{i({k}_{0}\left({x}_{m,n}^{^{\prime}}\mathit{sin}\left(\theta \right)\mathit{cos}\left(\varphi \right)+{y}_{m,n}^{^{\prime}}\mathit{sin}\left(\theta \right)\mathit{sin}\left(\varphi \right)\right)+{\varphi }_{m,n})},$$

where the ${k}_{0}$ is the free space wavenumber and ${I}_{m,n}$, ${x}_{m,n}^{^{\prime}}$ and ${y}_{m,n}^{^{\prime}}$ are amplitude, x coordinate, and y coordinate of *m,n*th element respectively. ${\varphi }_{m,n}$ is the phase of *m,n*th element of (M row* N column) reflectarray and the only parameter that can be changed or adjusted to scan the beam in electronically beam-scanning reflectarrays. The u–v mapping is needed to implement the angular transformation in array factor formulation, where $(u=\mathrm{sin}\left(\theta \right)\mathrm{cos}\left(\varphi \right), v=\mathrm{sin}\left(\theta \right)\mathrm{sin}(\varphi ))$. After applying this transformation, the array factor formula is changed to below:

2 $$AF(u,v){\sum}_{m=1}^{M}{\sum}_{n=1}^{N}{I}_{m,n}{e}^{i({k}_{0}\left({x}_{m,n}^{^{\prime}}u+{y}_{m,n}^{^{\prime}}v\right)+{\varphi }_{m,n})}.$$

### Fitness function and evaluating

**Figure 2:**
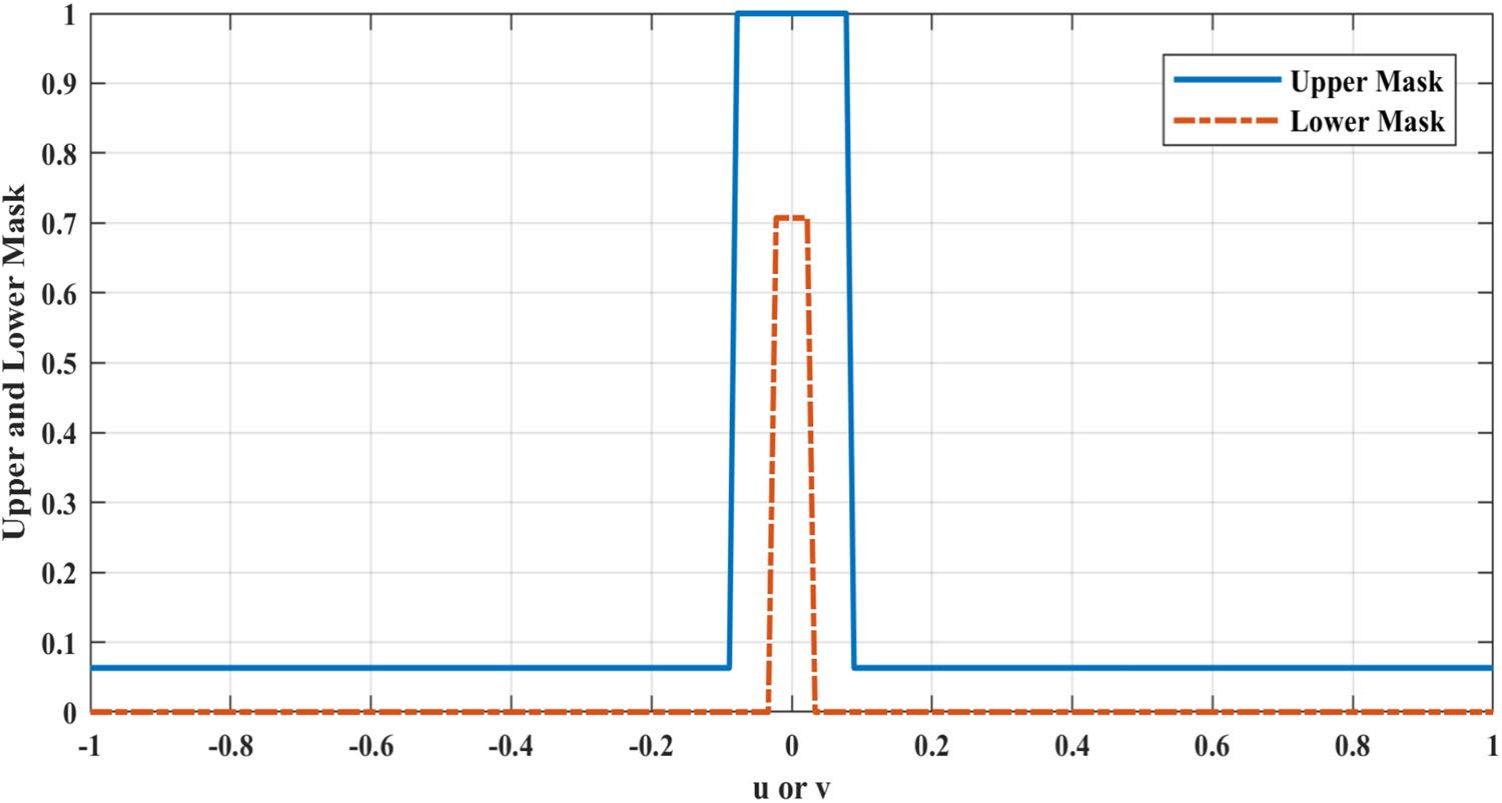
Upper and lower masks.

The fitness function in each optimization algorithm is one of the most important functions required for the optimization process because this function evaluates the value of the proximity of the optimal response to the desired response. The fitness function that is used to optimize the array factor of reflectarray is:

3 $$Fitness\left(u,v\right)=\left(U{M}^{2}-{\left|AF\right|}^{2}\right)*\left(L{M}^{2}-{\left|AF\right|}^{2}\right)+\left|U{M}^{2}-{\left|AF\right|}^{2}\right|*\left|L{M}^{2}-{\left|AF\right|}^{2}\right|,$$

where $UM$ and $LM$ are the upper and lower masks on the *u–v* plane respectively. The upper mask is defined to adjust the sidelobe level (SLL) and beam-width, and the lower mask is set to adjust the half-power beam-width (HPBW). Also, both of them adjust the direction of the main beam. These masks are used in both horizontal and vertical planes. In (3) all parameters are normalized and if at a specific point, the array factor (AF) is placed between the upper and lower masks, the value of the fitness function gets to zero (zero error). Otherwise, the value of the fitness function will be greater than zero with a positive amount. Figure 2 shows the upper mask and lower mask amplitude over *u* or *v*.

### Algorithm function and parameters setup

The functions used in GAPSO are given in Table 1. These functions are applied to the GA optimization part of the GAPSO algorithm.

**Table 1:** Functions types for GAPSO.

Functions	Type
Selection	Roulette wheel
Cross-over	Uniform
Selection for mutation	Random
Mutation	Random

The parameters of GAPSO are the sum of the GA and PSO parameters. The values of GAPSO parameters are expressed in Table 2. In this table $w$, $C1$ and $C2$ are the inertial weight and relative velocity weights of the PSO algorithm respectively, the values of these parameters are set to increase the convergence speed of the algorithm^31,32^.

**Table 2:** Values of GAPSO parameters.

Parameters	Value
Number of total populations	200
Number of PSO population	100
Number of GA population	100
Range of variables changes	[− 2π, 2π]
Range of variables speed change	[− 0.1π, 0.1π]
W	From 1 to 0.4 linear decreasing
C1	2
C2	2
Percent of cross-over	80
Percent of mutation	20

## Reflectarray setup

**Figure 3:**
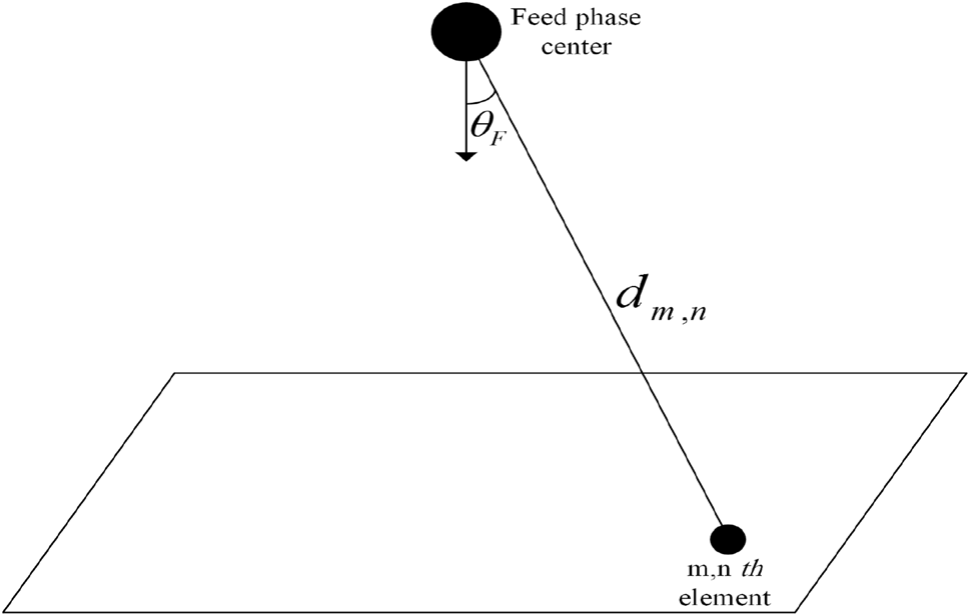
Feed and elements position in reflectarray.

**Figure 4:**
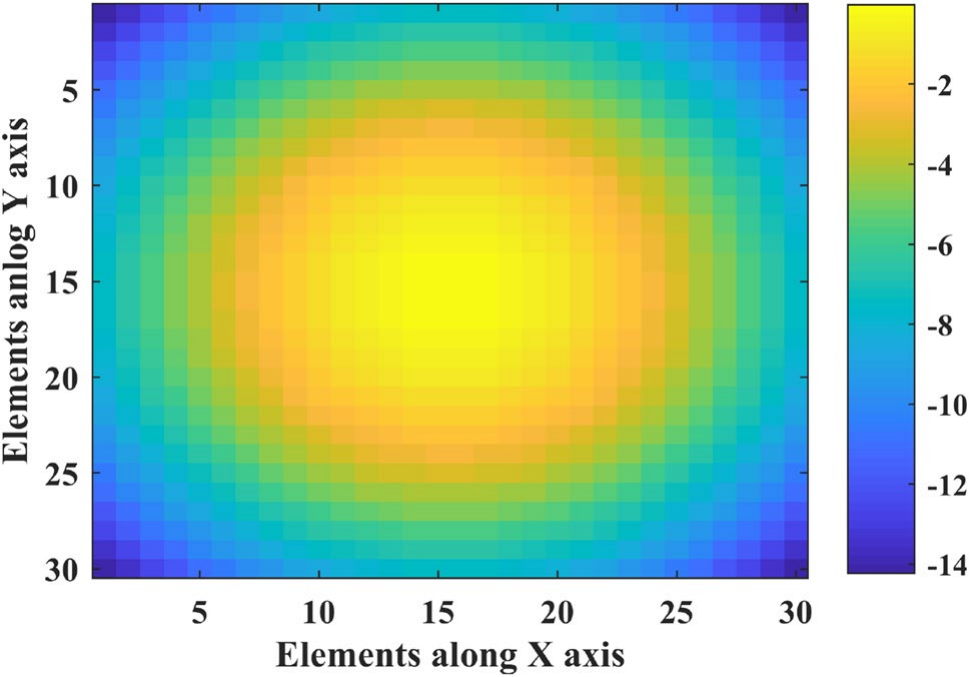
Normalized amplitude distribution on reflectarray (dB).

Reflectarray that used in this paper, contains a 30 × 30 (M = 30, N = 30) rectangular printed array and an axial symmetric feed with ${\mathrm{cos}}^{15}\left(\theta \right)$ radiation pattern model. Element spacing in the printed array is uniform and equals ${\lambda }_{0}/2$ at ${f}_{0}=10 \mathrm{GHz}$. The feed is pointed at the center of the printed array and perpendicular to it. And the distance between the center of the printed array and the phase center of the feed is $21.5{\lambda }_{0}$. There are no special requirements for the feed pattern, but if the feed pattern is symmetrically axial, the amplitude distribution over the array will be symmetrical. Also, the feed and array dimensions are effective in determining the feed distance from the center of the array for a proper compromise between illumination efficiency and spillover efficiency, the feed dimensions relative to the array dimensions will be effective in reducing the blockage effect^33^. By using (4) and Fig. 3, amplitude distribution on reflectarray is shown in Fig. 4.

4 $$\left|{E}_{Fn}\right|=\left(\frac{{A}_{0}}{{d}_{m,n}}\right){\mathrm{cos}}^{q}({\theta }_{F}).$$

In the above equation, $\left|{E}_{Fn}\right|$ is the amplitude of the electrical field that radiated from a source to the elements and *q* $=15$. ${d}_{m,n}$ is the distance between the phase center of the feed and the center of each element and ${A}_{0}$ is the constant value. To compensate the distance difference between the center of each element and phase center of the feed and to achieve beam in bore-sight of reflectarray without optimizing, each elements’ phase is calculated by using:

5 $${\varphi }_{m,n}=-{k}_{0}{d}_{m,n}.$$

**Figure 5:**
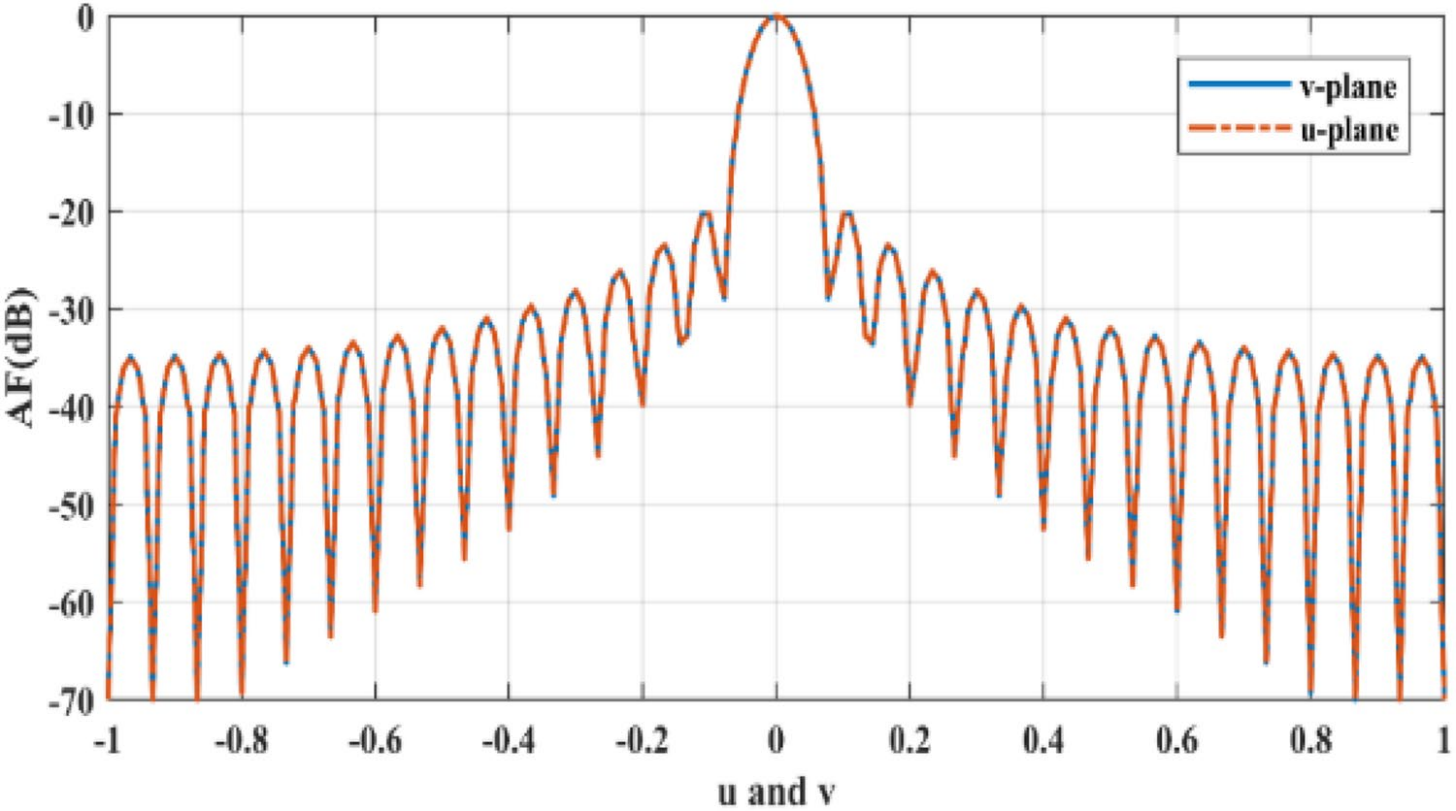
Bore-sight non-optimized normalized AF.

Figure 5 shows the bore-sight non-optimized normalized AF and as shown in this figure, SLL for the non-optimized array factor of the reflectarray is − 20 dB in the bore-sight beam direction.

## Novel phase distributions and optimization results

### Ordinary phase distribution

In the ordinary phase distribution used for all conventional electronically beam-scanning reflectarrays, the phase of all elements needs to be changed to displace the main beam at different angles. So, all the array elements need phase shifters except in arrays with an odd number of rows or columns. In large arrays (arrays with a large number of elements) the phase shifters cost and the complexities of implementation are the major disadvantages of this phase distribution. To overcome the mentioned drawbacks, two novel phase distributions are proposed in the following.

### Phase distribution with constant phase elements

**Figure 6:**
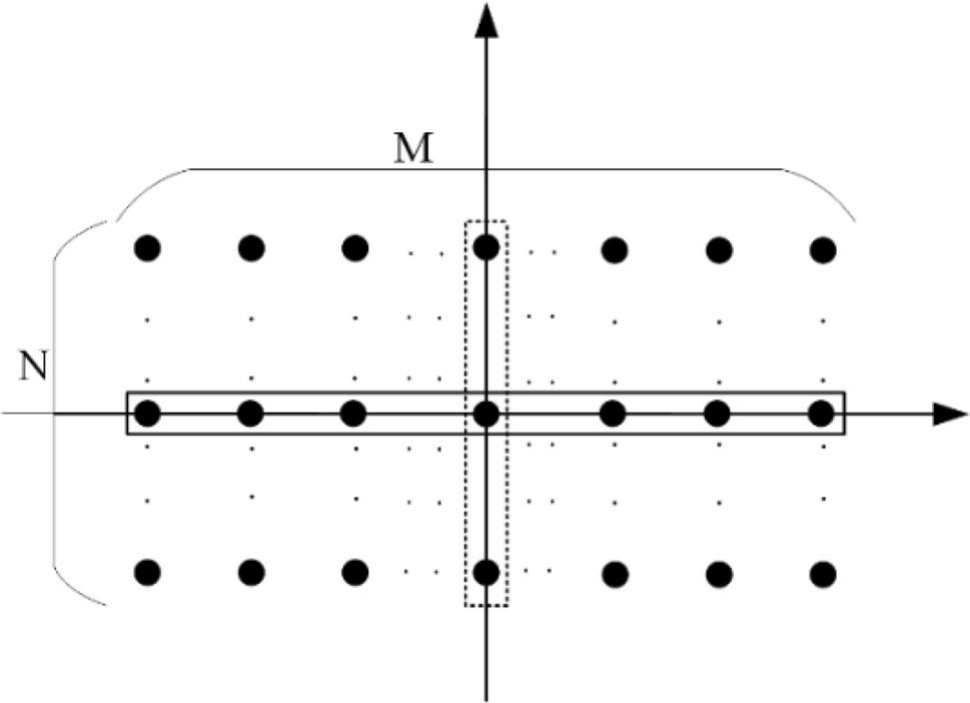
Array structure with an odd number of rows or columns.

**Figure 7:**
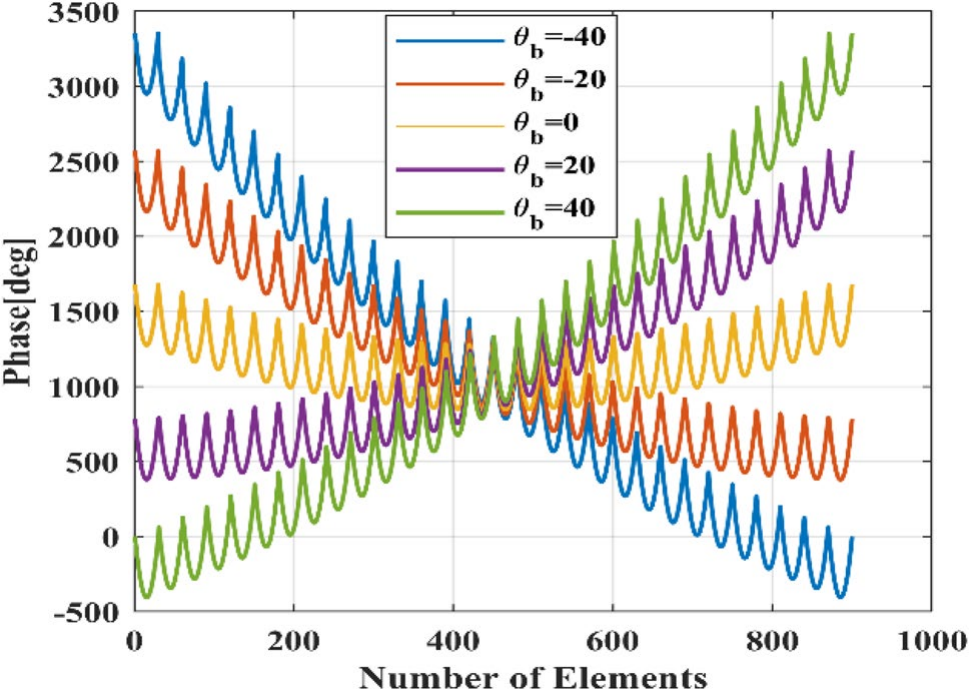
Analytical phase distributions for 30 × 30 reflectarray with different values of beam-direction.

Considering the array in Fig. 6 with an odd number of elements in the row (M) or column (N) and according to array theory, to scan the main beam in one of the principal planes (azimuth or vertical) there is no need to change the phase of elements in the middle row or column. So, these elements are constant phase elements in beam-scanning arrays. But in the reflectarrays with an even number of rows or columns, according to (6), there is no element that phase remains constant in the analytical phase synthesis method during the beam-scanning.

6 $${\varphi }_{R}\left({x}_{m,n}^{^{\prime}},{y}_{m,n}^{^{\prime}}\right)=-{k}_{0}\mathit{sin}\left({\theta }_{b}\right)\mathit{cos}\left({\varphi }_{b}\right){x}_{m,n}^{^{\prime}}-{k}_{0}\mathit{sin}\left({\theta }_{b}\right)\mathit{sin}\left({\varphi }_{b}\right){y}_{m,n}^{^{\prime}}+{k}_{0}{d}_{m,n},$$

where ${\varphi }_{R}\left({x}_{m,n}^{^{\prime}},{y}_{m,n}^{^{\prime}}\right)$ is the phase required for each element in the $\left({x}_{m,n}^{^{\prime}},{y}_{m,n}^{^{\prime}}\right)$ to achieve the beam direction at $({\theta }_{b},{\varphi }_{b})$. Figure 7 shows the non-periodical analytical phase distribution for mentioned array setup in “Reflectarray setup” for different beam directions in the *v-plane*, where elements are horizontally numbered in the array. As shown in this figure there are no elements that phase remain constant during the electronically beam-scanning.

Therefore, to implement the constant phase elements of the phased array with odd rows or columns, to reflectarrays, some elements have to be selected randomly from the mentioned array setup and considered as CPEs. CPEs have a constant phase during the beam-scanning and their phase will not be optimized or changed for all of the beam angles. CPEs’ phases are fixed to the values that compensate the distance difference between the center of the elements and phase center of the feed and calculated using (5). The number of CPEs is increased to such an extent that the specification of the optimized pattern using these elements in the reflectarray is approximately equal to the pattern synthesized by optimizing the phase of all elements. To change the beam direction in this type of phase distribution, only the phase of ordinary elements (OEs) (element could be changed or optimized) is optimized and changed. So, o these elements are the only ones need phase shifters and the other elements can be replaced with passive (without active phase shifter) unitcells.

### Phase distribution with CPEs and phase symmetry plane

Other drawbacks of one plane electronically beam-scanning reflectarrays with active phase shifters are the biasing device design complexity and expensive cost. One way to solve these problems is to use CPEs, as explained in the previous section. Another proposed method is to create symmetry in the phase of elements. If the CPEs and the symmetry phase plane are used together, costs and implementation complexities will dramatically decrease. For simultaneous use of the advantages of both methods, in addition, for selecting some elements as CPEs, the phase of all elements (CPEs and OEs) in the array as follows:

7 $${\varphi }_{m,n}={\varphi }_{m,N-n+1}.$$

In other words, according to Fig. 9, the phases of the element on the right side of the red line are equal to the phase of elements on the left side of that line.

### Optimizing and results

GAPSO is applied to the described reflectarray with explained phase distributions to find appropriate phase distributions for moving the main beam direction in the *v-plane,* and in the *u-plane,* the main beam direction remains in *u* = 0. In addition to beam-scanning in the v-plane, upper masks and lower masks are defined to fix the SLL below − 24 dB and remain the HPBW without any changes in both u and v plane compared to the non-optimized boresight beam shown in Fig. 5.

**Figure 8:**
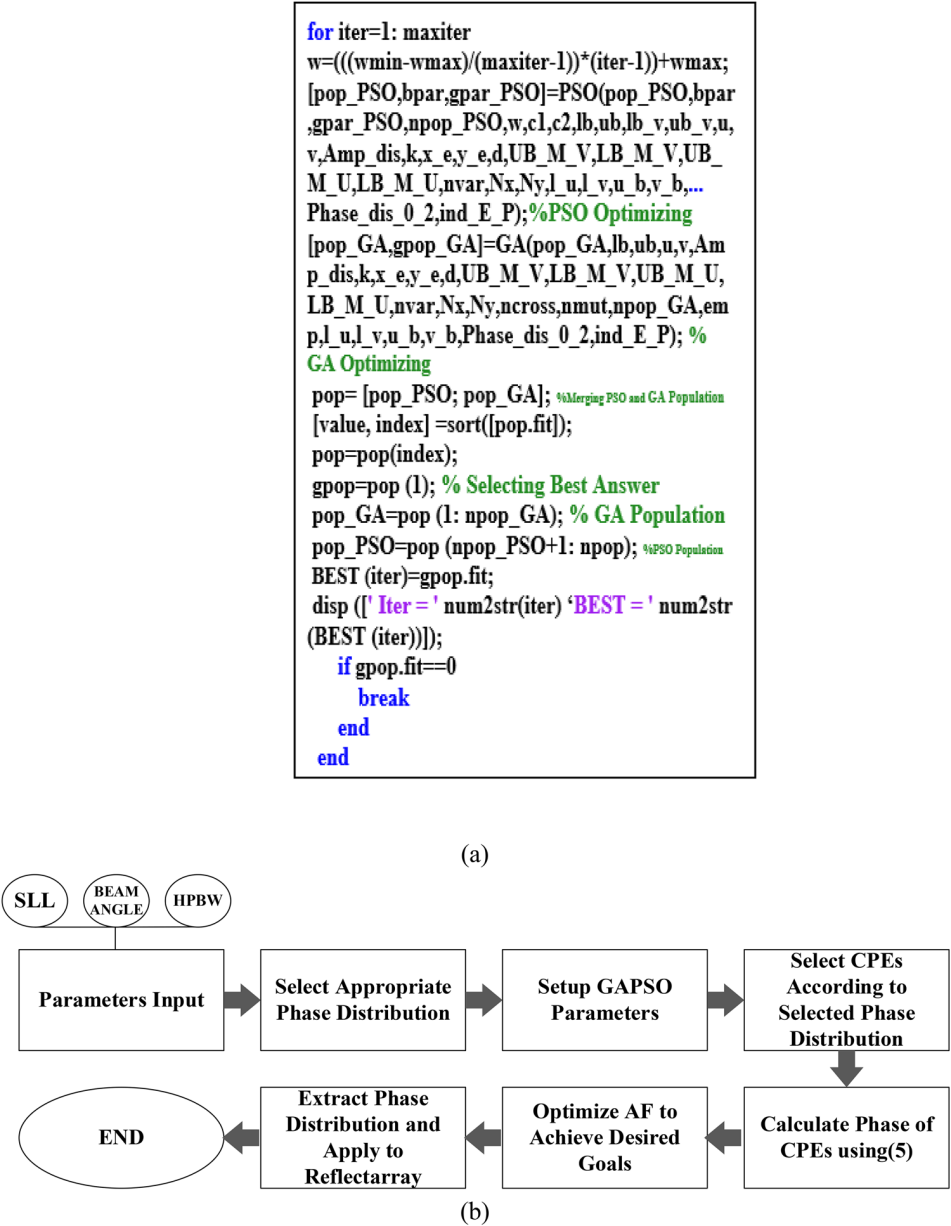
(**a**) AF and Phase distribution synthesis diagram. (**b**) The main loop of the MATLAB program.

**Figure 9:**
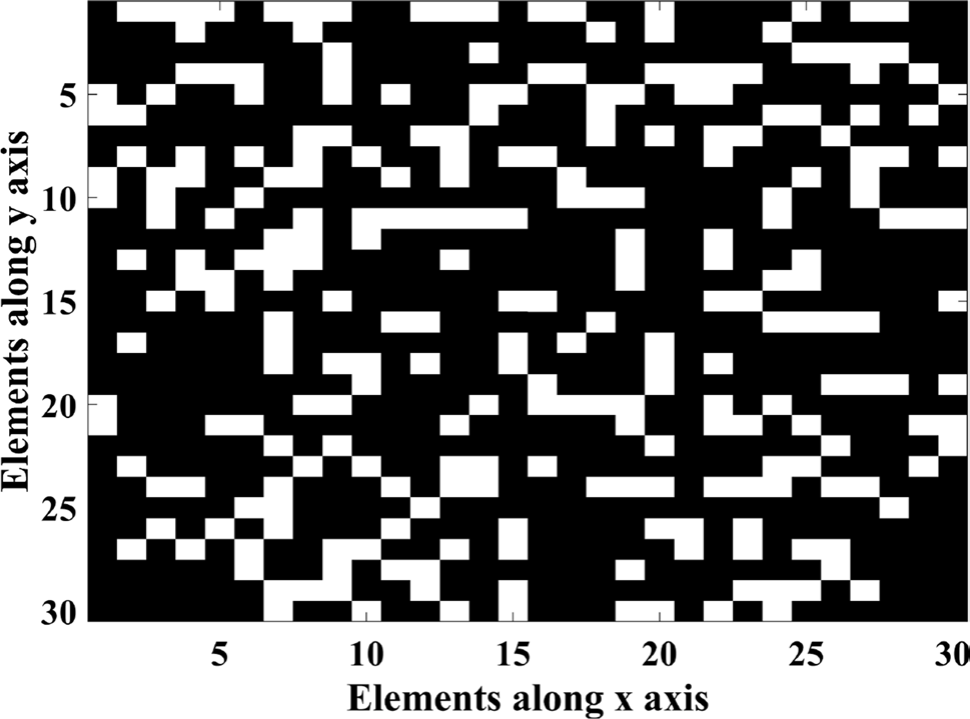
Array structure with only CPEs.

The AF of reflectarray is optimized in both u and v planes simultaneously and the total fitness value is the sum of the fitness values in u plane and v plane. Figure 8a shows the AF and Phase distribution synthesis diagram and Fig. 8b shows the main loop of optimizing program in MATLAB. Also, to scan the main beam from ${\theta }_{b}=-40^\circ$ to ${\theta }_{b}=40^\circ$ with almost 3 dB AF gain reduction through the phase distribution with only CPEs, the 260 elements are the maximum possible number that can be randomly selected as CPEs. The white elements shown in Fig. 9 are the CPEs and the black colored elements are the OEs. To change the beam direction in this type of phase distribution, only the phase of OEs (71.2% of total elements) could be changed and optimized during bam-scanning. So, only these elements need active phase shifters.

**Figure 10:**
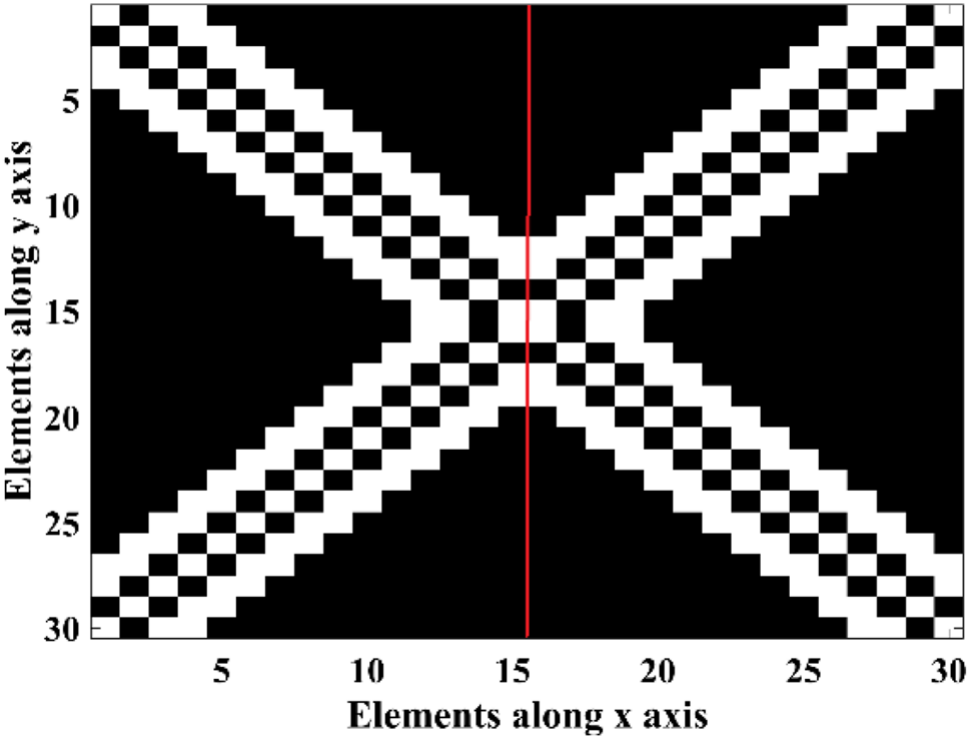
Array structure with CPEs and Phase symmetry plane.

To implement phase distribution with CPEs and phase symmetry plane and desired beam scanning conditions in *v-plane*, CPEs must be selected symmetrical in array combination, one of the best choices for CPEs shape is the symmetric diagonal shape. Also, the phase symmetry plane is applied to the explained array setup. Figure 10 demonstrated the array structure used in this kind of phase distribution. White-colored elements are the CPEs and black-colored elements are OEs.

**Figure 11:**
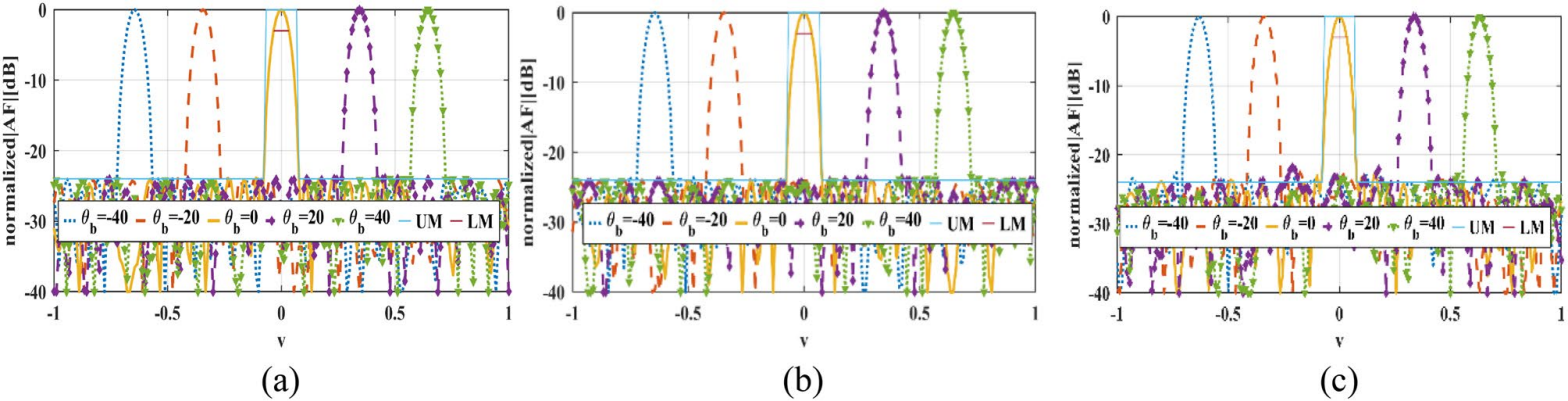
Optimized normalized AF in *v-plane* for (**a**) Ordinary phase distribution. (**b**) Novel phase distribution with CPEs and (**c**) Novel phase distribution with CPEs and phase symmetry plane.

**Figure 12:**
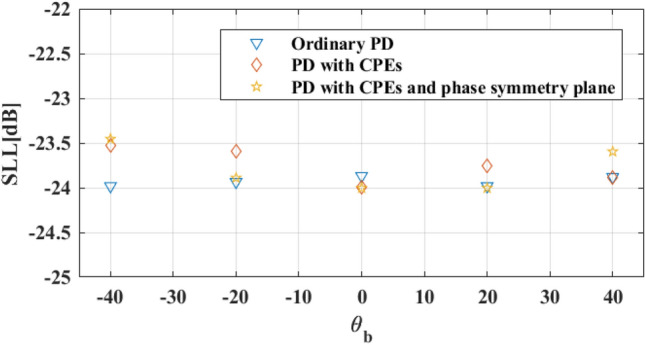
SLL (dB) over different values of beam direction in *v-plane* for phase distributions.

**Figure 13:**
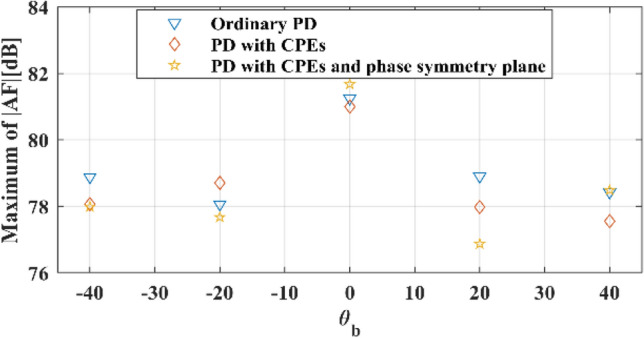
Maximums of $\left|AF\right|$ over different values of beam direction in *v-plane* for phase distributions.

Because of the symmetric phase of all elements and 260 CPEs, only 35.6% of all existing elements in the described reflectarray setup are optimized, and the phases of other elements are either constant (CPEs) for all beam angles or equal to the other elements. The normalized *v-plane* reflectarray AF patterns for described phase distributions and array setup are shown in Fig. 11. As shown in this figure, the main lobe of the reflectarray AF pattern is moved from ${\theta }_{b}=-40^\circ$ to ${\theta }_{b}=40^\circ$ in *v-plane* for all of the phase distributions. The SLLs of patterns for all of the phase distributions are nearly or below − 24 dB, as illustrated in Fig. 12. And HPBWs of them are not significantly changed and it is almost equal to non-optimized pattern. Figure 13, shows the maximum $\left|AF\right|$ over different values of beam direction in *v-plane* for three explained phase distributions. As shown in this figure, maximums of $\left|AF\right|$ for these phase distributions are close, and almost 3 dB AF gain reduction is achieved for 80° beam-scanning range.

**Figure 14:**
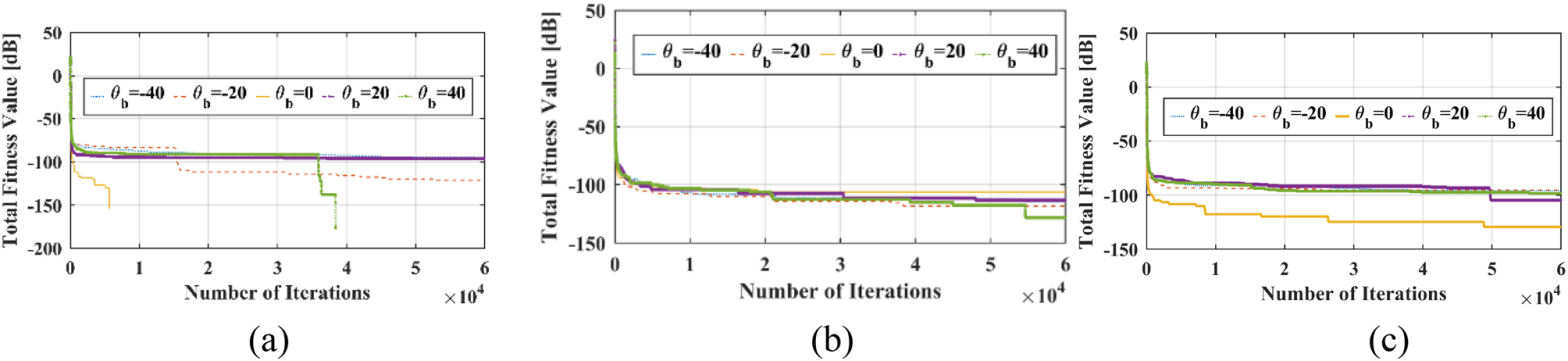
Total fitness function over iteration for (**a**) Ordinary phase distribution. (**b**) Novel phase distribution with CPEs and (**c**) Novel phase distribution with CPEs and phase symmetry plane.

**Figure 15:**
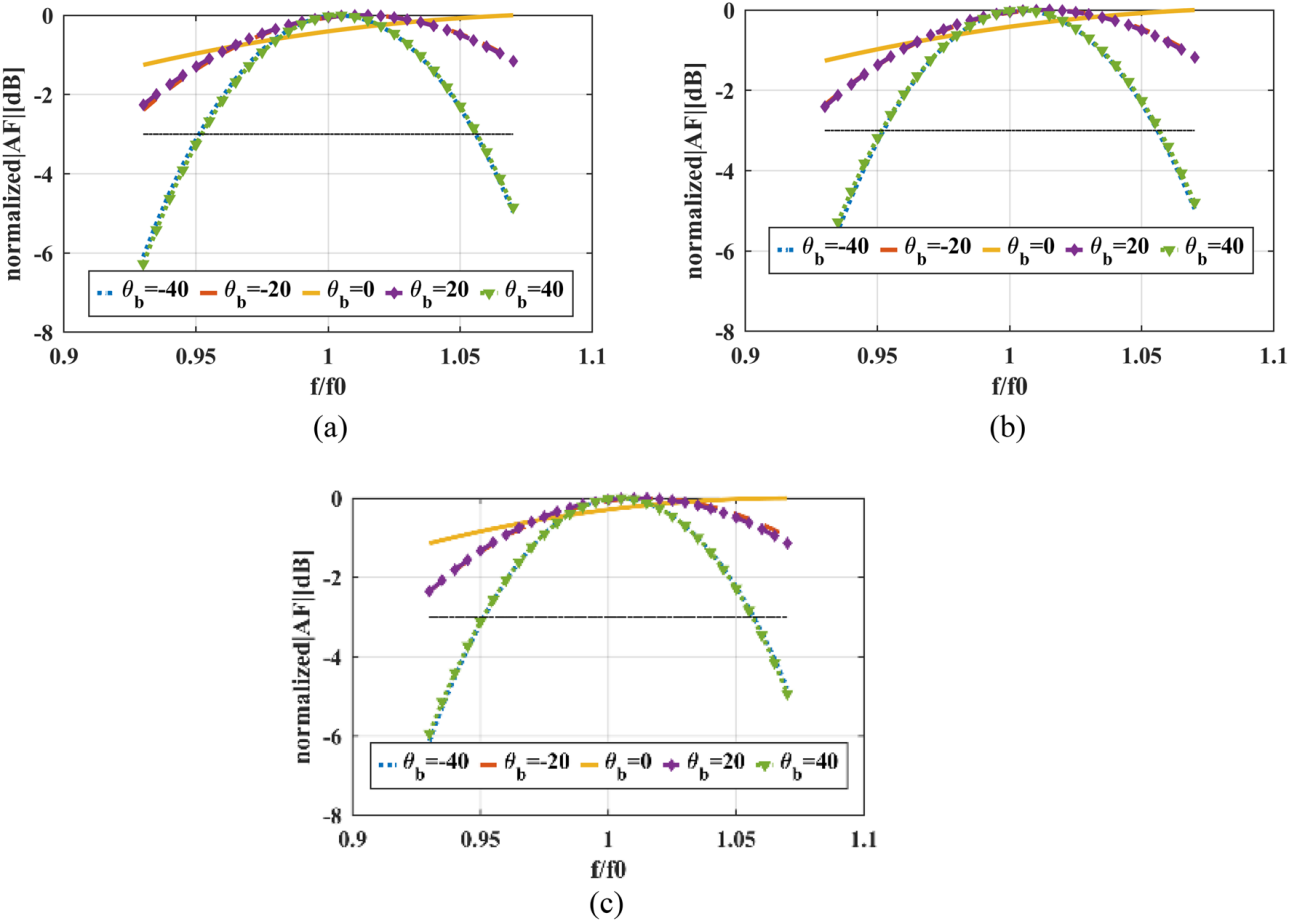
Optimized normalized AF in v plane over normalized frequency (f/f0) for (**a**) Ordinary phase distribution. (**b**) Novel phase distribution with CPEs and (**c**) Novel phase distribution with CPEs and phase symmetry plane.

The total fitness values over different iterations of the optimization process for all phase distributions are demonstrated in Fig. 14. Also it shows that the values of total fitness for all phase distributions and all beam directions are nearly zero. Hence, the reflectarray AF optimized patterns in both *u* and *v-plane* are meeting the optimization goals. Figure 15 shows Optimized normalized AF in v plane over normalized frequency (f/f0) for three described phase distributions. In all three phases distributions, the patterns in ${\theta }_{b}=-40^\circ$ and ${\theta }_{b}=40^\circ$ are the limiting factors for frequency bandwidth and the pattern in these beam angles decreases rapidly over frequency in comparison to the other beam angles. The optimization is done for the only center frequency of reflectarray, next the frequency bandwidths of phase distributions for 3 dB reduction in AF amplitude for the desired beam-direction are extracted and calculated.

So, according to Fig. 15, to achieve 80° beam-scanning range with only 3 dB $\left|AF\right|$ compression over frequency, the fractional bandwidth is almost 11% for all three-phase distribution. It means that for mentioned fractional bandwidth all parameters such a beam-with and SLL remain valid and only HPBW and gain of reflectarray will be reduced.

## Simulation results

**Figure 16:**
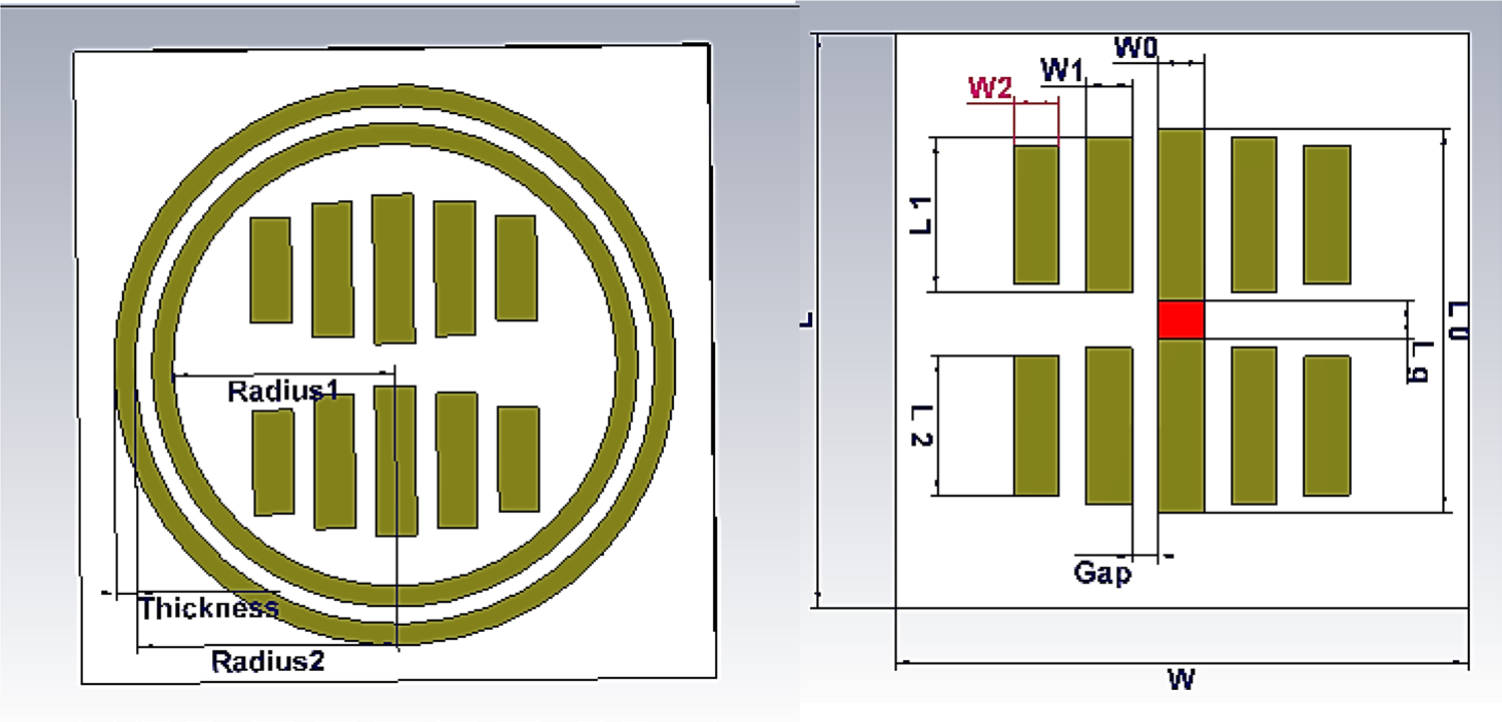
Structure of unitcell.

**Figure 17:**
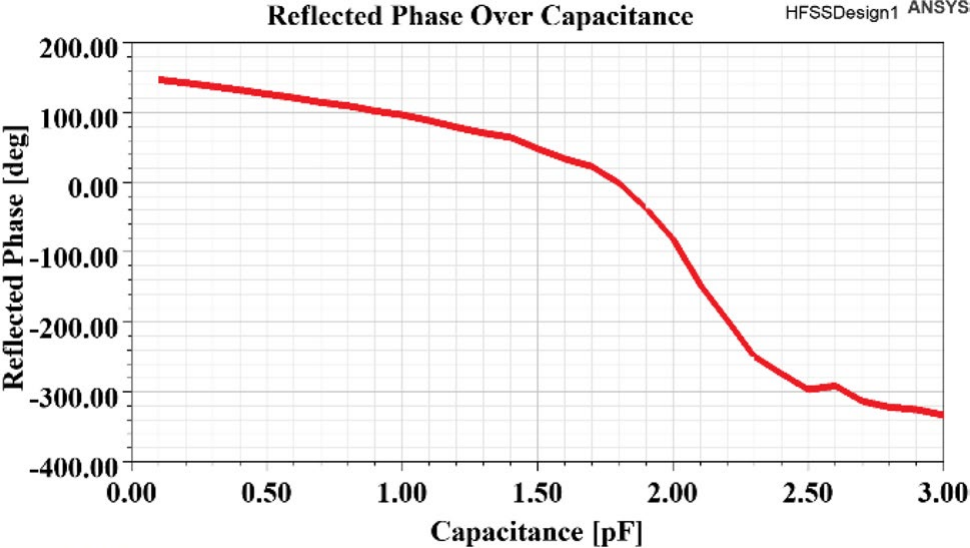
Reflected phase versus capacitance change of unitcell.

To verify the proposed PDs, a 10 × 10 reflectarray’s phase distribution has been optimized to change the main beam direction in the *v-plane.* To implement the mentioned reflectarray, first, a unitcell was designed. The outline of the unitcell, as shown in Fig. 16, consists of an arrangement of electrical dipoles in the form of a yagi-uda antenna that are symmetrical about the center of the cell. A voltage variable capacitor (varactor diode) is placed in the middle of the unitcell which is responsible for shifting the reflected phase of the cell. As shown in Fig. 17. The phase of the reflected wave at a central frequency of 10 GHz has a phase shift range of more than 450°. The values of unitcell parameters are listed in Table 3.

**Table 3:** Parameters of unitcell.

Parameter	Value
Frequency	10 GHz
Substrate	RO4003 (32mil)
W = L	15 mm
L00	$\left(\frac{L0}{2}\right)-\left(\frac{Lg}{2}\right)$
L1	r1 × L00
L2	r2 × L00
r1	0.9
r2	0.7
W0 = W1 = W2	0.95 mm
Radius1	5.25 mm
Radius2	6.15 mm
Thickness	0.5 mm
L0	8.1 mm
Gap	0.5 mm
Gap between rings	0.4 mm

**Figure 18:**
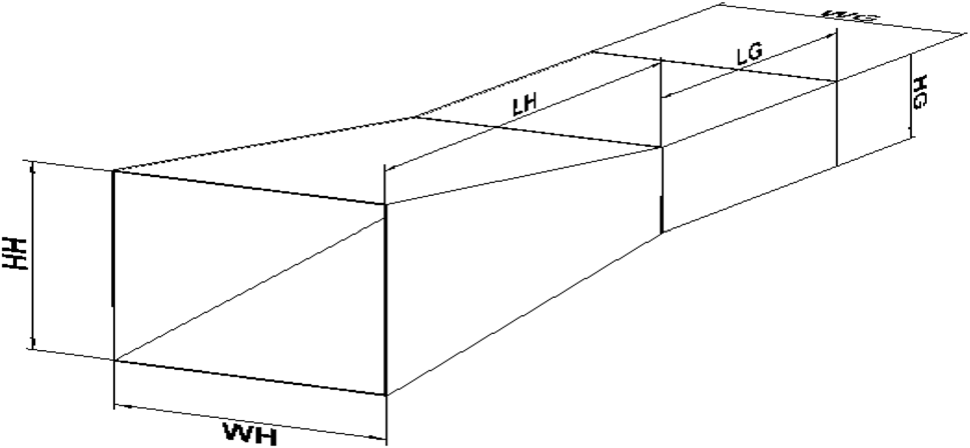
Structure of the feed horn.

**Figure 19:**
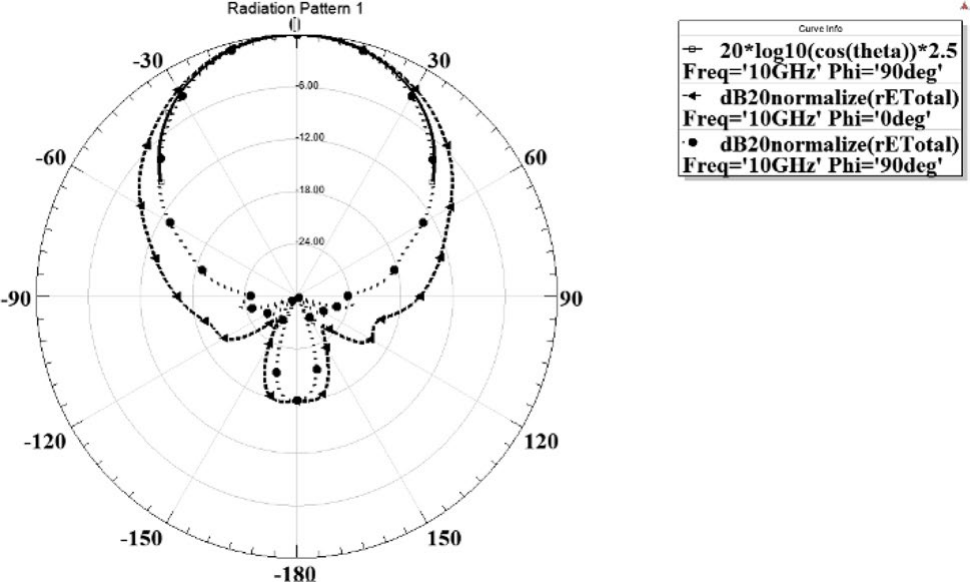
The simulated pattern of the feed.

In the next step, a pyramidal horn is designed as a feed of the reflectarray. The structure of the feed is shown in Fig. 18 and the dimensions of this feed are listed in Table 4. And it is simulated in ANSYS HFSS. The simulated pattern of the horn is shown in Fig. 19. As shown in this figure the pattern can be modeled as ${\mathrm{cos}}^{2.5}\left(\theta \right)$ in both principal planes of the feed, so $q=2.5$ for this reflectarray. Also, the pattern of the feed is almost the axial symmetric pattern. Consequently, this pyramidal horn is suitable to be used as a reflectarray feed.

**Table 4:** Dimensions of the feed horn.

WG	22.9 mm
HG	10.2 mm
LG	29.98 mm
WH	25.75 mm
HH	26.229 mm
LH	47.775 mm

**Figure 20:**
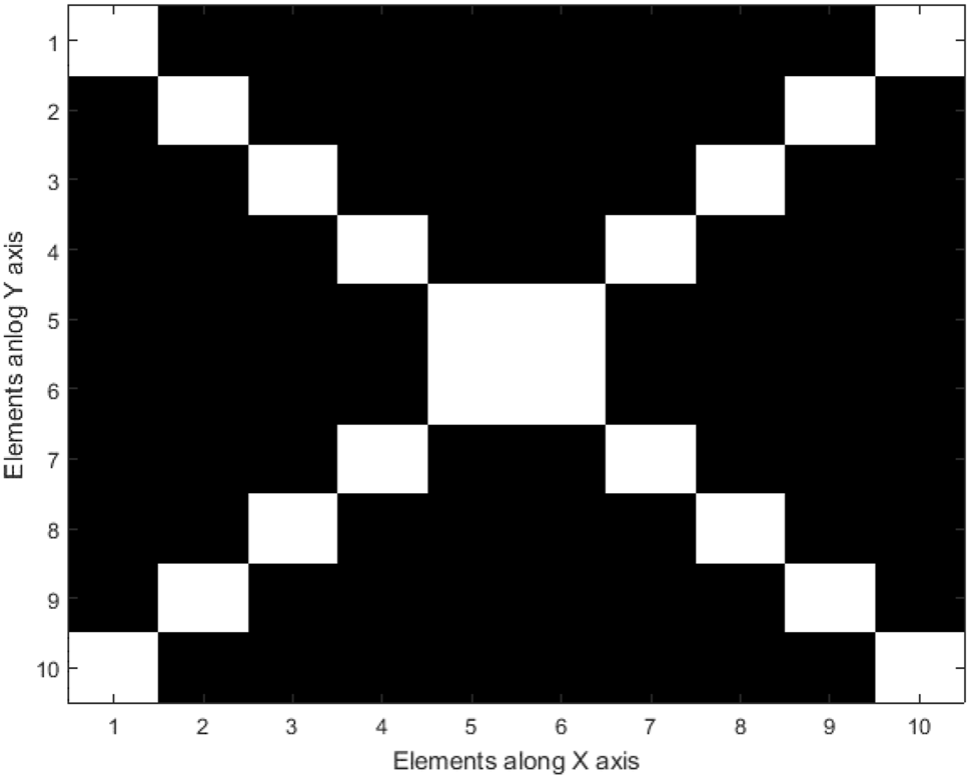
Elements in 10 × 10 reflectarray.

The phase distribution with CPEs and phase symmetry plane is selected as a phase distribution type for reflectarray. The phase distribution of reflectarray has been optimized to change the main beam direction of reflectarray between ${\theta }_{b}=-10^\circ$ to ${\theta }_{b}=10^\circ$ in v-plane with $SLL\le -19 \mathrm{dB}$. The GAPSO is used to optimize the phase distribution. The distance between the phase center of the feed and the center of the reflectarray is $3.5\lambda$. Figure 20 shows the elements in the reflectarray, white-colored elements are CPEs, and the phase symmetry plane as explained before exists. In this reflectarray 20 elements have been chosen to be CPEs. For these elements, the proposed unitcell only has the constant value capacitor in the middle, for obtaining the value of the capacitor first the phase of these elements is calculated as explained before next, the phase translated to the capacitor value using Fig. 17. For the other elements, the varactor diode is placed in the middle of the unitcell, but because of the phase symmetry plane, the biasing device is only needed to bias half of the total numbers of them. Also, because of this plane, it is enough to simulate only half of the reflectarray.

**Figure 21:**
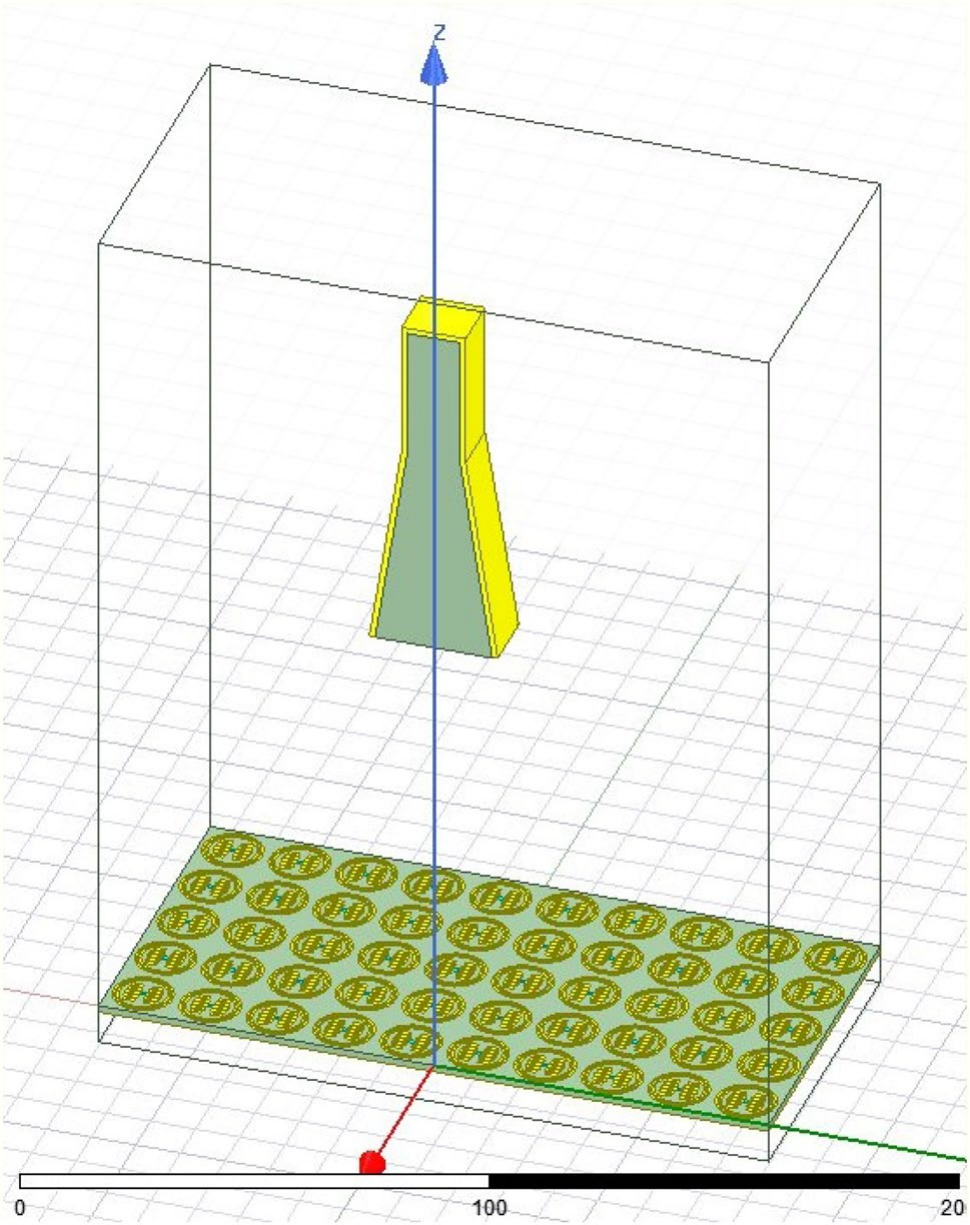
Reflectarray in ANSYS HFSS.

**Figure 22:**
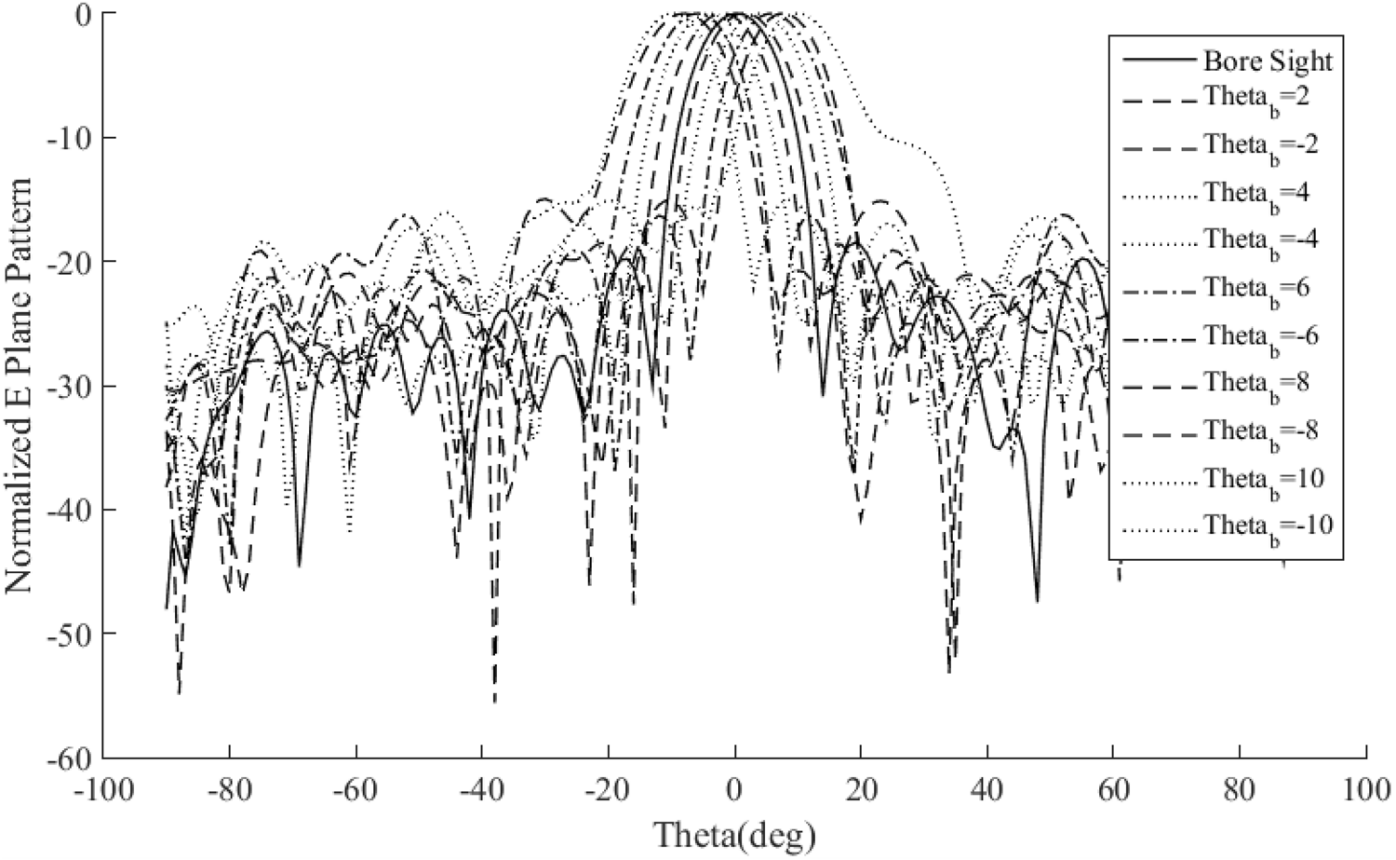
Normalized pattern of 10 × 10 reflectarray.

Figure 21 shows the half of the reflectarray in the ANSYS HFSS, and Fig. 22 shows the simulated normalized pattern of the reflectarray. As shown in this figure the beam-scanning range is achieved using the proposed method.

As mentioned before, in all previous works with electronically beam-scanning reflectarrays, all unitcell of the array had an active phase shifter even for scanning the beam in only one principal plane. All unitcells in those types of reflectarrays needed this active phase shifter, such as a PIN diode, a varactor diode, etc. In this paper, the need for active phase shifters for a large number of elements is eliminated and the phase of these elements remains unchanged at all angles of beam scanning, and these elements can be replaced with passive elements without active phase shifters.

In Table 5 the comparison between the proposed beam-scanning reflectarray and the previous electronically beam-scanning reflectarrays are listed. The third column of the table denotes the portion of the unitcells with active phase shifter in reflectarray, these phase shifters make the reflectarray reconfigurable. The smaller the number of these unitcells in the reflectarray, the lower the cost and complexity of implementing large reflectarrays. The fourth column in that table shows the portion of unitcells with active phase shifter that biased or switched independently of another unitcells, by reducing the number of these unitcells in the reflectarray, implementation of the biasing device or control unit of the reflectarray will be low cost and easy. From the table, it is verified that for electronically beam-scanning reflectarrays, the phase distribution with CPEs and phase symmetry plane with CPEs that proposed in this paper, was first used in the phase distribution of electronically beam-scanning reflectarrays.

**Table 5:** Comparison table.

References	Unitcell Combination for beam scanning	The portion of active unitcells in reflectarray	The portion of unitcells with independent phase shifter	Phase synthesis method
^17^	Active	100%	100%	Analytical
^9^	Active	100%	100%	Optimization
^10^	Active	100%	Not mentioned	Optimization
^34^	Active	100%	100%	Analytical
This work	Active and Passive	71.2% (CPEs) 71.2%(CPEs and symmetry plane)	71.2% (CPEs) 35.6%(CPEs and symmetry plane)	Hybrid algorithm optimization

## Conclusion

Microstrip beam-scanning reflectarrays phase distribution synthesis methods have been studied in this paper. Hybrid GAPSO has been described for optimizing the phase distribution on reflectarrays. In addition to ordinary phase distributions, two novel phase distributions with constant phase elements and symmetry phase planes have been proposed to achieve beam-scanning capability in reflectarrays. The array factors of 30 × 30 reflectarray by using these three kinds of phase distributions have been optimized to displace the beam in the vertical plane from $-40^\circ$ to 40° The optimized result shows that GAPSO has a good performance to optimize the phase distribution of beam-scanning reflectarrays.

Results obtained from optimizing two novel phase distributions demonstrates that for beam-scanning reflectarrays, it is not necessary to optimize the phase of all elements and by optimizing just a few numbers of them (71.2% of total elements in phase distribution with CPEs and only 35.6% in phase distribution with CPEs and phase symmetry plane) the reflectarray will have the same performance in Frequency bandwidth, Gain and Side Lobe Level compared to the ordinary phase distribution or optimizing phase of all elements. Hence, due to the absence of phase shifters in a large number of elements in these two novel phase distributions, the cost and complexities of designing and implementation of electronically beam-scanning reflectarray and its biasing devices will be decreased.
